# Whole-Genome Sequence Dataset of *Rhodococcus qingshengii* IEGM 267—Terpenoid Biotransformer Toward Genetic Functional Annotation

**DOI:** 10.3390/molecules31173083

**Published:** 2026-09-02

**Authors:** Polina Y. Maltseva, Natalia A. Plotnitskaya, Irina B. Ivshina

**Affiliations:** Perm Federal Research Center, Ural Branch of the Russian Academy of Sciences, 13a Lenin Str., 614990 Perm, Russia; inbox.98@bk.ru (P.Y.M.); luchnikova.n@mail.ru (N.A.P.)

**Keywords:** whole-genome sequencing, terpenoids, *Rhodococcus*, CYP450

## Abstract

**Background/Objectives**: Microbial biotransformation of monoterpenoids is a promising approach for obtaining bioactive compounds. *Rhodococcus* species are attractive biocatalysts due to their metabolic versatility and ability to transform hydrophobic substrates. In this study, we investigated the catalytic potential of *Rhodococcus qingshengii* IEGM 267 toward carveol isomers and explored genomic features that may underlie this activity. **Methods**: The strain was cultivated in mineral medium supplemented with (−)-*trans*-carveol. Biotransformation products were analyzed by TLC and GC–MS. The draft genome was sequenced, assembled, taxonomically assigned, and annotated using standard bioinformatics tools. **Results**: *Rhodococcus qingshengii* IEGM 267 efficiently converted (−)-*trans*-carveol to carvone. Genome analysis confirmed the taxonomic assignment of the strain and revealed a large repertoire of oxidoreductases, including monooxygenases, hydroxylases, and dehydrogenases. Seven genes encoding cytochrome P450-dependent oxygenases were identified as candidate enzymes potentially involved in carveol oxidation. **Conclusions**: *R. qingshengii* IEGM 267 is an efficient and stereoselective biocatalyst for (−)-*trans*-carveol oxidation. The results of bioinformatics analysis suggest an alternative enzymatic basis for this transformation and provide a foundation for future functional characterization.

## 1. Introduction

At present, the search for efficient and selective biocatalysts for the conversion of plant-derived terpenoids remains highly relevant for biotechnology and pharmaceutical research. One of the most promising areas in pharmacology is the synthesis of compounds with potential pharmacological effects from accessible and inexpensive raw materials, such as plant terpenoids [1,2]. Among these, monoterpenoid (−)-carveol is of particular interest as starting material for new bioactive substances and promising substrates for target derivatives [3]. Bacterial transformation of monoterpenoids enables highly stereo- and regioselective modification of precursor molecules, yielding bioavailable and bioactive derivatives in a single biotechnological step.

Among terpene-transforming microorganisms, actinomycetes of the genus *Rhodococcus* occupy a special position due to their broad metabolic capabilities and exceptional tolerance to hydrophobic substrates [4]. These bacteria are known to catalyze the oxidation and modification of a wide range of mono-, di-, and triterpenoids, making them promising candidates for the discovery of new biotransformation pathways and enzymes [5].

**Figure 1:**
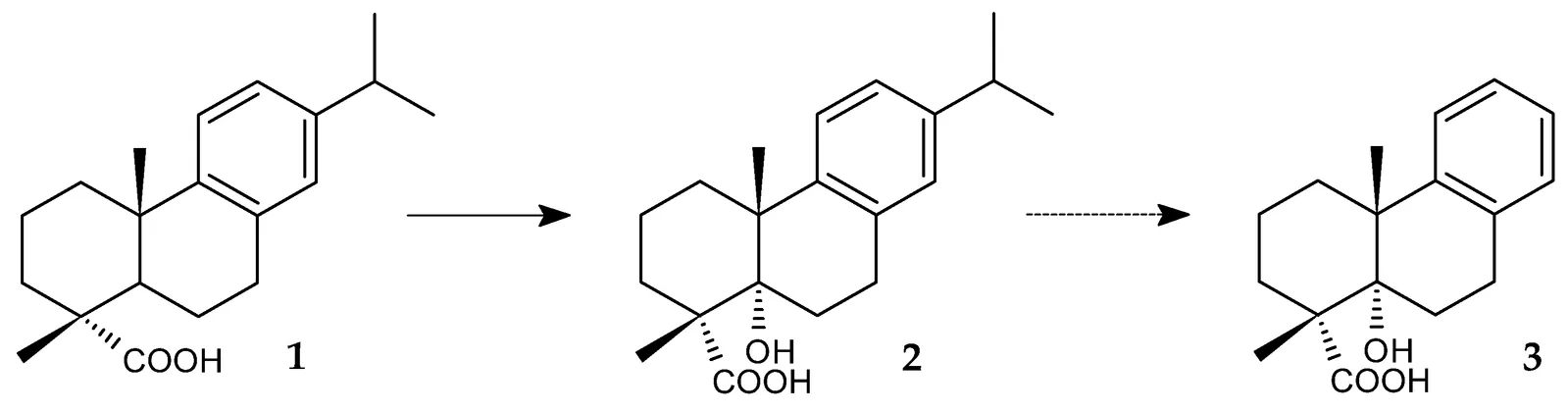
Scheme of dehydroabietic acid (**1**) biotransformation by *Rhodococcus qingshengii* IEGM 267; **2**—5α-hydroxy-abieta-8,11,13-triene-18-oat; **3**—15,16,17-trinor-abietane-type compound [6].

In this context, the strain *Rhodococcus qingshengii* IEGM 267, isolated from oil-polluted soil in the Perm region, is of particular interest. Previously, this strain was shown to bioconvert high concentrations (500 mg/L) of the diterpenoid dehydroabietic acid and to form previously unidentified metabolite 5α-hydroxy-abieta-8,11,13-triene-18-oat (**2**) and 15,16,17-trinor-abietane-type compounds (**3**) (Figure 1) [6]. However, its ability to transform monoterpenoids and the genetic basis of this activity have not yet been sufficiently characterized.

The aim of this study was to evaluate the catalytic potential of *R*. *qingshengii* IEGM 267 and to identify the candidate genes involved in (−)-*trans*-carveol transformation using bioinformatics tools.

## 2. Results and Discussion

### 2.1. Biotransformation Activity

**Figure 2:**
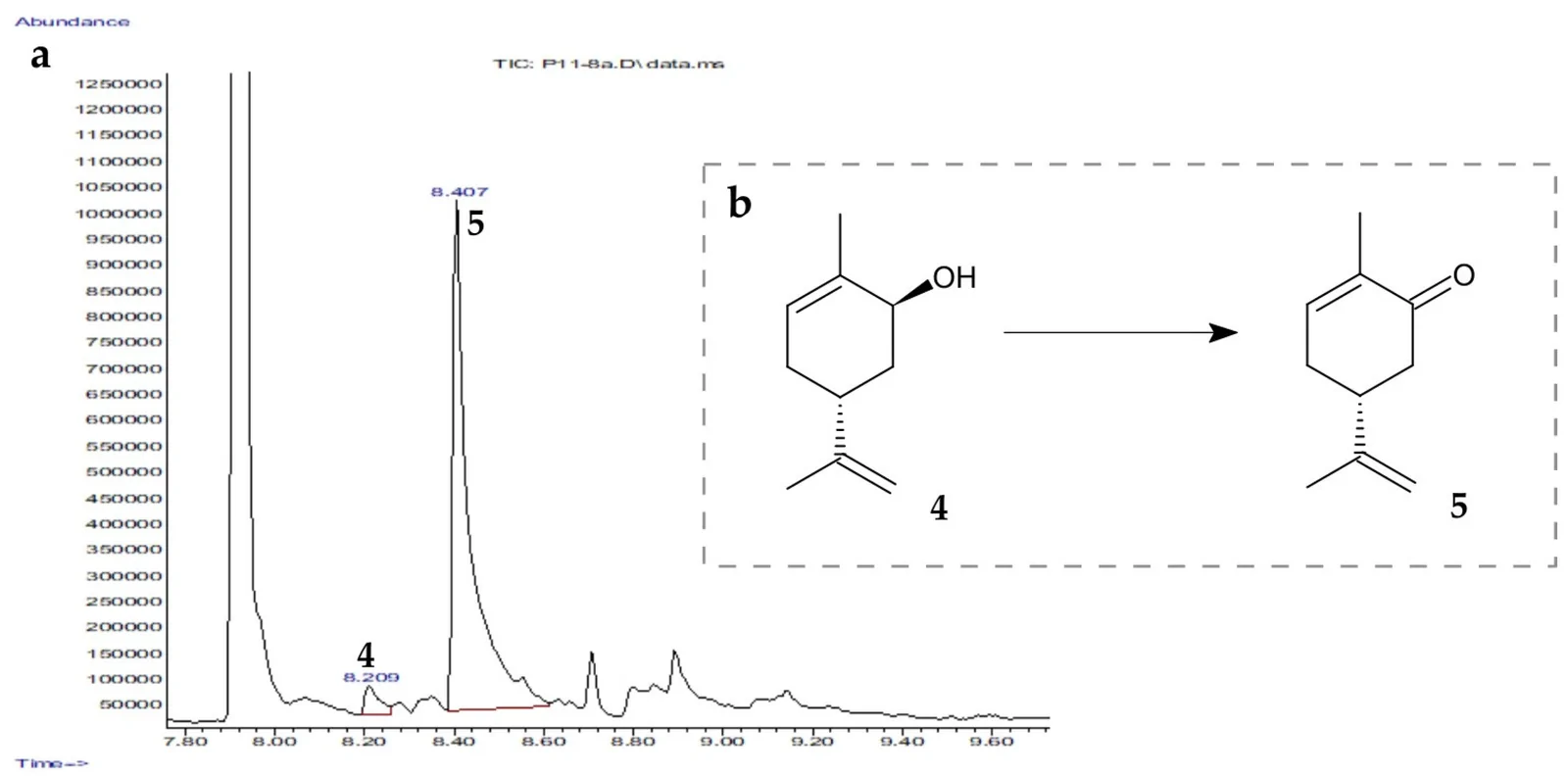
Biotransformation of (–)-*trans*-carveol by *Rhodococcus qingshengii* IEGM 267: (**a**)—chromatogram of the extract obtained during biotransformation; (**b**)—scheme of biotransformation. **4**—(–)-*trans*-carveol; **5**—carvone.

We investigated the transformation activity of *Rhodococcus qingshengii* IEGM 267 toward the plant-derived monoterpenoid (–)-*trans*-carveol. TLC and GC–MS analyses revealed that efficient bioconversion (95% yield) was achieved after 7 days of cultivation with (–)-*trans*-carveol (**4**) (Figure 2). The primary product was identified as carvone (**5**), a valuable compound widely utilized in the perfume industry and recognized for its antimicrobial, anti-inflammatory, and antitumor properties [2].

This stereospecific oxidation aligns with previously reported microbial transformations of carveol. For instance, *R*. *erythropolis* DCL14 has been shown to oxidize carveol to carvone [7] via the action of carveol dehydrogenase (CDH), encoded by the *limC* gene [8], with further stereospecific degradation. In *R. opacus* PWD4 cells, CDH catalyzed the hydroxylation of *D*-limonene to *trans*-carveol, while carvone formation occurred only as a minor product and strongly depended on the growth substrate and cultivation conditions [9]. In our study, *R. qingshengii* IEGM 267 showed an efficient conversion of (−)-*trans*-carveol to carvone, which was not further transformed, suggesting that the oxidation may proceed via an alternative pathway.

To elucidate the molecular determinants underlying this stereospecific transformation, we performed a bioinformatics analysis of the *R*. *qingshengii* IEGM 267 genome, with a focus on *limC* orthologs and associated monoterpenoid catabolism gene clusters.

### 2.2. Bioinformatics Analysis

The assembled genome of *R*. *qingshengii* IEGM 267 comprised 231 contigs, totaling 7.2 Mbp in length, with an N50 of 173,729 bp, a GC content of 62.3%, and an average sequencing coverage of 84.4×. Genome annotation identified 7212 coding sequences (CDSs) and 56 RNA genes (Table 1).

**Figure 3:**
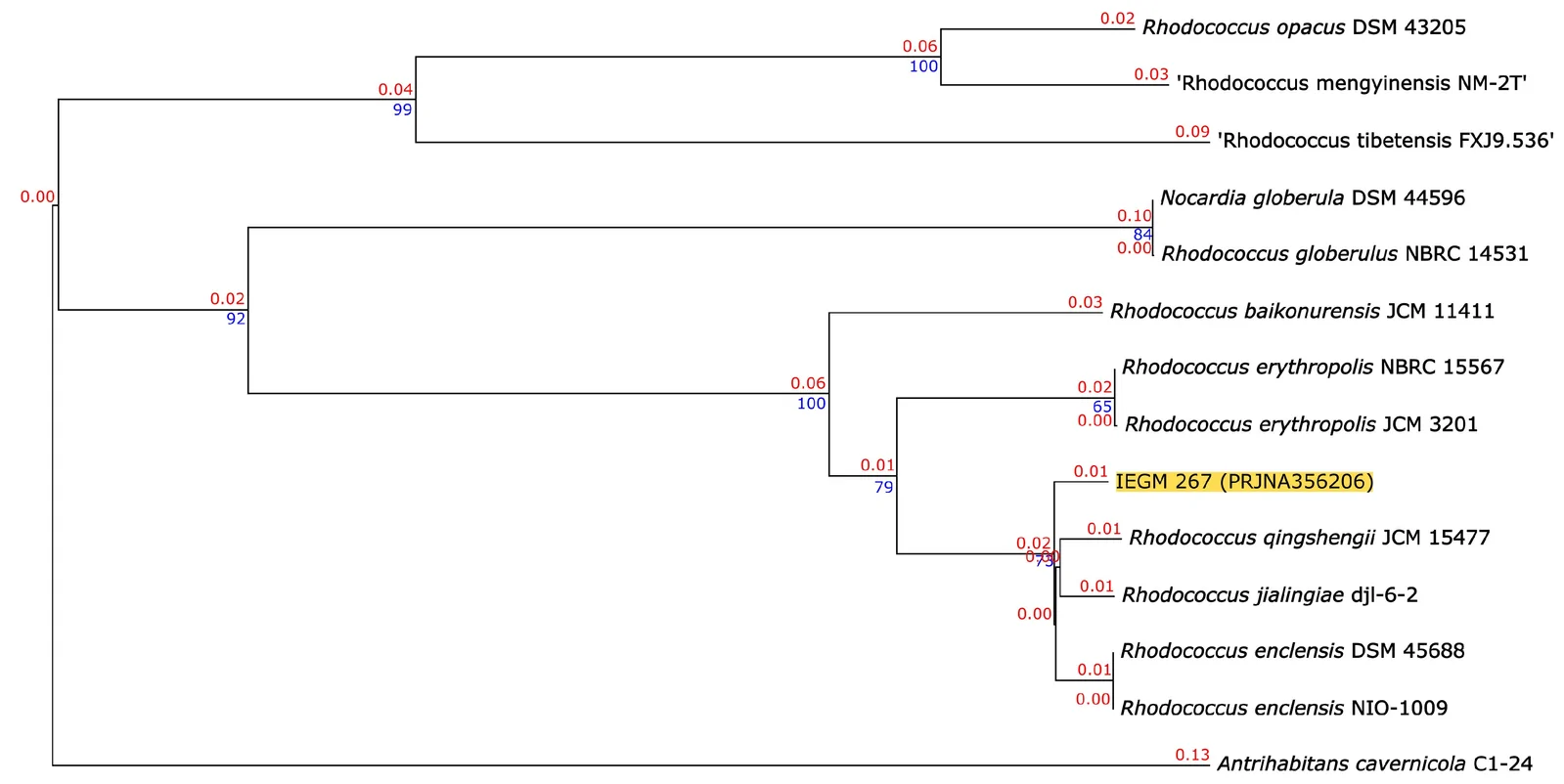
Phylogenetic tree of genomes of *R. qingshengii* IEGM 267 and type strains from TYGS database. Tree inferred with FastME 2.1.6.1 [10] from GBDP distances calculated from genome sequences. The branch lengths are scaled in terms of GBDP distance formula *d_5_*. The numbers above branches are GBDP pseudo-bootstrap support values >60% from 100 replications, with an average branch support of 70.3%. The tree was rooted at the midpoint [11].

Using phenotypic methods (Table S1) and genetic analysis (Table 2), the IEGM 267 strain was identified as belonging to the *R. qingshengii* species (Figure 3). The resulting species and subspecies clusters are presented in Table S2. The clustering yielded eight species clusters and the provided query strains were assigned to one of these. The dDDH scores were more than 70% in comparisons of the IEGM 267 genome with genomes of *R. jialingiae*, *R*. *enclensis* and *R*. *qingshengii*-type strains and less than 70% in comparisons with genomes of *R*. *erythropolis*. The ANI scores were above 94–95% for all four species. According to the List of Prokaryotic names with Standing in Nomenclature (LPSN), the names *R. jialingiae*, *R*. *enclensis* and *R*. *erythropolis* are heterotypic synonyms for *R*. *qingshengii*, validly published under the International Code of Nomenclature of Prokaryotes (ICNP) (https://lpsn.dsmz.de/species/rhodococcus-qingshengii, accessed on 15 April 2026).

**Figure 4:**
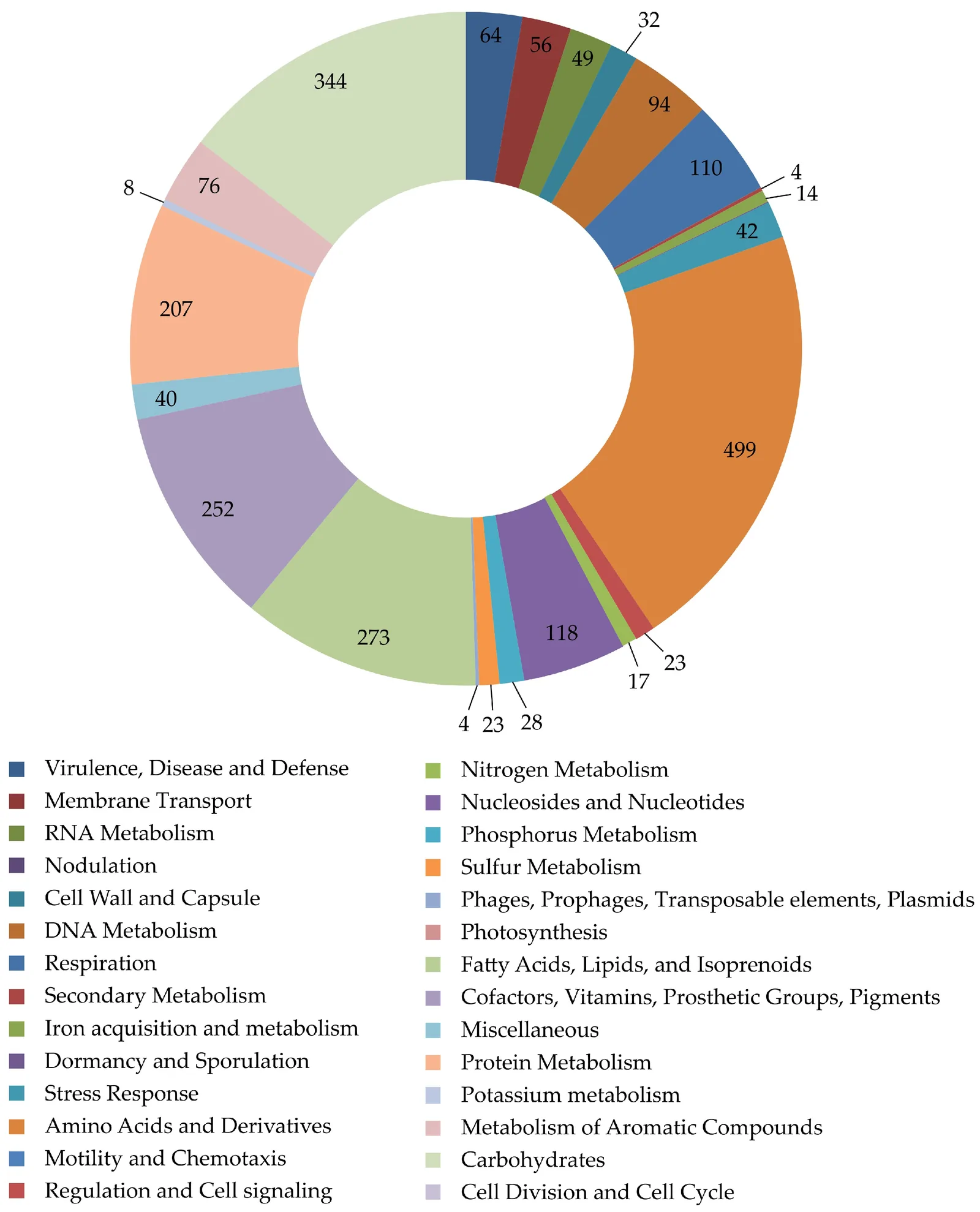
Distribution of subsystem categories in genome of *R*. *qingshengii* IEGM 267. Image obtained using SEED Viewer 2.0.

Among 7212 CDSs, genes of unknown and known functions were present in a 80:20 ratio. The functional category distribution of the known genes is shown in Figure 4. Notably, we identified five genes encoding enzymes of the nonmevalonate pathway for isoprenoid biosynthesis, including EC 2.2.1.7, 1.1.1.267, 2.7.7.60, 2.7.1.148 and 4.6.1.12. Genome mining using AntiSMASH revealed two biosynthetic gene clusters associated with terpene biosynthesis (Table 3).

Using genomic analysis of *R*. *qingshengii* IEGM 267 we identified 76 genes encoding monooxygenases, 41—dioxygenases, 11—hydroxylases, and 386—dehydrogenases. According to data from other researchers, the conversion of carveol by rhodococci is catalyzed by the enzyme carveol dehydrogenase (CDH), encoded by the *limC* gene [8]. However, the results of our RAST annotation analysis revealed that this gene is absent from the genome of strain IEGM 267. Furthermore, no other known genes involved in terpenoid conversion were found, except for two genes encoding limonene-1,2-epoxide hydrolase (EC 3.3.2.8). A BLASTP search of the proteome of *R*. *qingshengii* IEGM 267 using the experimentally characterized CDH sequence (UniProt accession Q9RA05) as a query did not identify a recognizable homolog (Table S4). Nevertheless, we identified seven genes encoding cytochrome P450-dependent oxygenases, which may represent potential homologs or analogs of the *limC* gene (Table 4). Additionally, one gene encoding lanosterol 14α-demethylase, one gene encoding cholesterol oxidase (EC 1.1.3.6), three genes encoding steroid C27-monooxygenase (EC 1.14.13.141), four genes encoding 3-ketosteroid-9α-monooxygenase oxygenase component (EC 1.14.13.142), one gene encoding steroid C26-monooxygenase (EC 1.14.13.221), and five genes encoding 3-oxosteroid 1-dehydrogenase (EC 1.3.99.4) were identified.

After identifying seven genes encoding cytochrome P450s in the genome of *R*. *qingshengii* IEGM 267, we performed a detailed analysis of their genetic surroundings to identify potential functional partners, regulatory elements, and signatures of horizontal gene transfer. This approach not only confirmed that these genes belong to active metabolic clusters but also allowed us to infer their possible biological roles.

Analysis of the genetic surroundings of the cytochrome P450 genes revealed their organization into functionally specialized clusters, characterized by the presence of oxidative–reductive metabolism genes, transporters, and transcriptional regulators (Table 5). In five out of seven cases, directly upstream of the P450 genes are regulators of the AcrR family, indicating a likely induction of expression in response to aromatic compounds or xenobiotics. Gene No. 6 is associated with the regulator HxlR, which responds to aldehydes, and gene No. 7 with the regulator MerR, involved in the detoxification of heavy metals. The most complete set of redox partners, including ferredoxin reductase, 2Fe-2S ferredoxin and short-chain dehydrogenase, was found in the gene No. 3 cluster confirming its putative catalytic activity. Functional analysis of neighboring genes showed that these P450s were involved in different metabolic pathways: degradation of fatty acids and xenobiotics, carnitine metabolism and siderophore biosynthesis, arsenic detoxification and oxidation of polyprenylphenols. The presence of mobile elements and restriction or modification genes located in the vicinity of the five and seven genes suggests that these loci may have been transferred horizontally, which is in accordance with the known plasticity of the *Rhodococcus* genomes under conditions of stress. The findings of this study indicate that *R*. *qingshengii* IEGM 267 possesses a diverse array of P450 cytochromes suited for the degradation of multiple organic compounds and can thrive in contaminated environments, highlighting its potential for biotechnological applications.

Pairwise alignment of the amino acid sequences of the P450 cytochromes identified in *R*. *qingshengii* IEGM 267, both with each other and with CDH from *R*. *erythropolis* (UniProt Q9RA05) [8], showed a very low degree of identity, not exceeding 32% (Figure S2). This indicates that the identified genes likely encode previously undescribed P450 enzymes. Such data also suggest that the biotransformation pathway of (–)-*trans*-carveol in the IEGM 267 strain differs from previously characterized mechanisms.

Subsequently, for each of the identified CYP450 genes, a search for identical amino acid sequences was conducted in the UniProt database (Table 6). Most of the identified proteins are orthologs or homologs of CYP450, widely distributed among representatives of the genus *Rhodococcus* and closely related taxa *Gordonia*, *Williamsia*, and *Nocardia*. Moreover, according to annotations, many of the identified proteins are involved in the metabolism of sterols and steroids.

In the context of this work, the results obtained for CYP450 No. 3 are of particular interest. The amino acid sequence of this enzyme is apparently highly conserved, as evidenced by its 100% identity with P450 cytochromes from *R*. *kyotonense* and *R*. *cerastii*, and, in particular, with linalool 8-monooxygenase (EC 1.14.13.151) from *R*. *fascians*. These data suggest that CYP450 No. 3 may be involved in the transformation of terpenes and, in particular, participate in the conversions of (–)-*trans*-carveol.

Although our bioinformatics analysis provides valuable insights into the genetic basis of (–)-*trans*-carveol biotransformation, several limitations should be considered when interpreting the results. Functional annotations are based on homology searches against reference databases, which, despite their extensive coverage, may not fully capture the functional diversity of enzymes. A large proportion of the predicted CDSs are annotated as hypothetical or of unknown function, reflecting current gaps in database coverage and the limited availability of experimentally characterized proteins from closely related strains. Therefore, some genes actually involved in carveol oxidation may remain unannotated in our dataset. Moreover, the actual contribution of each candidate enzyme to the observed biotransformation cannot be deduced from genomic data alone. Consequently, targeted experimental validation, including gene knockout, transcriptomic profiling, heterologous expression, and in vitro enzymatic assays, is essential to conclusively assign function to the identified candidates. Despite these limitations, our study provides a robust genomic foundation for future functional characterization and expands the repertoire of terpenoid-transforming rhodococci.

## 3. Materials and Methods

### 3.1. Culture

*R*. *qingshengii* IEGM 267 was isolated from oil-polluted soil (Perm region, Russia) and deposited in the Regional Specialized Collection of Alkanotrophic Microorganisms (acronym IEGM, WFCC number 285, WDCM number 768). The strain uses hydrocarbons and crude oil as a sole carbon source, transforms dehydroabietic acid, forms cholesterol oxidase and is resistant to Cr^6+^ (5.0 mM), VO^2+^ (12.5 mM), VO_4_^3−^ (50.0 mM), and VO_3_^−^ (250.0 mM) (http://iegmcol.ru/strains/rhodoc/qingsh/r_qingsh267.html, accessed on 16 April 2026).

### 3.2. Cultivation Conditions

Culture was grown during 7 days in mineral salt medium RS containing (g/L): K_2_HPO_4_—2.0; KH_2_PO_4_—2.0; KNO_3_—1.0; (NH_4_)_2_SO_4_—2.0; NaCl—1.0; MgSO_4_—0.2; CaCl_2_—0.02; FeCl_3_×7H_2_O—0.01; and trace element solutions according to Postgate (0.1% *v*/*v*) [12]. The medium was supplemented with (–)-*trans*-carveol (*trans*-*p*-menta-6,8-dien-2-ol, C_10_H_16_O, CAS 1197-07-5) to a final concentration of 0.025% *v*/*v* and yeast extract (0.1 g/L) (FBIS SRCAMB, Obolensk, Russia).

### 3.3. Extraction and Analysis of Residual (–)-Trans-Carveol and Its Derivatives

To extract the residual (–)-*trans*-carveol and its derivatives, an equivalent volume of ethyl acetate was added to the cell suspension acidified with 10% HCl solution; after separation of the resulting emulsion, the ethyl acetate fraction was taken. Ethyl acetate was added to the remaining aqueous fraction for re-extraction; the procedure for separation and selection of fractions was repeated twice. The ethyl acetate fractions were combined and evaporated on a rotary evaporator Laborota 4000 (Heidolph, Schwabach, Germany). The residue was successively washed with a 1% NaHCO_3_ aqueous solution and distilled water (up to pH 7.0) and dried over anhydrous Na_2_SO_4_. The washed residue was used for analyses. Qualitative analysis was carried out by thin-layer chromatography (TLC) on Alugram^®^ Xtra SIL G/UV254 plates (Macherey-Nagel, Düren, Germany) in the *n*-hexane-ethyl acetate (1:1) system. Separated components were detected after drying and keeping the plates in iodine vapor.

Separation of extracts was performed by column chromatography using silica gel (SiO_2_; 60–200 µm; Macherey-Nagel, Düren, Germany) as carier and hexane:EtOAc from 100:0 to 0:100 as eluent.

The (–)-*trans*-carveol and its derivatives were analyzed using GC–MS data using a Agilent 7890A chromatograph (Santa Clara, CA, USA) with a quadrupole mass spectrometer Agilent 5975C as a detector, HP-5MS quartz column, 30,000 × 0.25 mm, and He (1 atm) as carrier gas. The column temperature was programmed from 50 to 280 °C. MS were recorded in the range of 50–700 *m*/*z* and compared with those from the NIST08 Library.

### 3.4. Whole-Genome Sequencing

For DNA extraction, the *R*. *qingshengii* IEGM 267 cells were grown in LB broth at 28 °C and 160 rpm for 28 h. Genomic DNA was isolated using a DNeasy PowerSoil Kit (Qiagen, Hilden, Germany). The shotgun library was prepared using a Nextera XT DNA Library Preparation Kit (Illumina, San Diego, CA, USA) according to the manufacturer’s instructions and sequenced on Illumina HiSeq 2500 instrument at Genotek, Moscow, Russia (https://www.genotek.ru/). The draft genome was assembled with SPAdes v. 3.9.

### 3.5. Bioinformatics Analysis

The genome sequence data were uploaded to the Type (Strain) Genome Server (TYGS), a free bioinformatics platform available under https://tygs.dsmz.de (accessed on 16 April 2026), for a whole-genome-based taxonomic analysis [13]. The analysis also made use of recently introduced methodological updates and features [14,15]. Information on nomenclature, synonymy and associated taxonomic literature was provided by TYGS’s sister database, the List of Prokaryotic names with Standing in Nomenclature (LPSN, available at https://lpsn.dsmz.de) [14,15]. Digital DNA–DNA hybridization (dDDH) and confidence intervals were calculated using GGDC 4.0 (Genome-to-Genome Distance Calculator 4.0, https://ggdc.dsmz.de/ggdc.php#, accessed on 16 April 2026). Identity (ANI) was determined using the EZBioCloud ANI calculator (https://www.ezbiocloud.net/tools/ani, accessed on 16 April 2026) [16].

The search for the candidate genes involved in terpene biotransformation, along with rRNA genes, was performed using the RAST (Rapid Annotation using Subsystem Technology, https://rast.nmpdr.org/, accessed on 15 April 2026) by the annotation scheme RASTtk with an automatic error fixing, frameshift correction, and similarity computation [17]. Target amino acid sequence comparisons were performed using the BLAST and Align tools available on the UniProt website (https://www.uniprot.org/, accessed on 13 July 2026) [18]. Biosynthetic gene cluster prediction was conducted using antiSMASH version 8.0.4 with the following settings: detection strictness relaxed; extra features used were KnownClusterBlast, SubClusterBlast, MIBiG cluster comparison, ActiveSiteFinder, RREFinder, and TFBS analysis (https://antismash.secondarymetabolites.org/, accessed on 15 April 2026). An amino acid sequence of experimentally characterized CDH protein from *R*. *erythropolis* (UniProt Q9RA05) was used as a query in search against the *R*. *qingshengii* IEGM 267 proteome. The search was conducted using a BLASTP.

## 4. Conclusions

The present study demonstrated that *Rhodococcus qingshengii* IEGM 267 is an efficient and stereoselective biocatalyst for the oxidation of (–)-*trans*-carveol to carvone, achieving a high conversion yield after 7 days of cultivation. Genome sequencing and annotation confirmed the taxonomic assignment of the strain and revealed a metabolically versatile genome enriched in oxidoreductases, including monooxygenases, hydroxylases, dehydrogenases, and seven cytochrome P450-dependent oxygenases. Notably, no recognizable CDH homolog was identified in the predicted proteome, suggesting that carveol oxidation in this strain is mediated by an alternative enzymatic system.

Bioinformatics analysis of the genomic context of the identified CYP450 genes indicated that several of them are embedded in functionally relevant clusters associated with redox metabolism, transport, and regulation, which supports their potential involvement in terpene transformation. Among these candidates, CYP450 No. 3 appears to be the most promising enzyme for future functional characterization because of its high sequence conservation with terpene-associated monooxygenases and its likely role in monoterpenoid metabolism. Overall, the data expand current knowledge of *Rhodococcus*-mediated terpene biotransformation and provide a basis for further experimental validation of the enzymes responsible for carveol oxidation.

## Figures and Tables

**Table 1:** Genome features for *R*. *qingshengii* IEGM 267 assembly (according to RAST).

Feature	Value
Size, bp	7,184,113
GC content, %	62.3
N50, bp	173,729
L50	14
Number of contigs	231
Number of CDSs	7212
Number of RNAs	56
Genome coverage	84.4×

**Table 2:** Overall genomic characteristics of *R*. *qingshengii* IEGM 267 compared with type strains in TYGS.

Subject Type Strain	dDDH (d_4_), %	G + C Content Difference, %	ANI, %
*R. jialingiae* djl-6-2	89.6	0.1	98.81
*R*. *enclensis* DSM 45688	89.0	0.01	98.72
*R*. *enclensis* NIO-1009	88.9	0.01	98.72
*R*. *qingshengii* JCM 15477	88.4	0.05	98.63
*R*. *erythropolis* JCM 3201	62.5	0.07	95.34
*R*. *erythropolis* NBRC 15567	62.5	0.08	95.36

**Table 3:** Biosynthetic gene clusters in the genome of *R*. *qingshengii* IEGM 267.

Coding Element	Number of Clusters
Non-ribosomal peptide synthetase	14
Non-ribosomal peptide metallophores	2
Type I Polyketide synthase	2
Terpene	2
Redox-cofactor	1
(Thio)azol(in)e-containing peptides	1
Beta-lactone containing protease inhibitor	1
Ectoine	1
Class III lanthipeptides	1
Unspecified ribosomally synthesized and post-translationally modified peptide product	1
RRE-element-containing cluster	1
Butyrolactone	1

**Table 4:** Genes of *R*. *qingshengii* IEGM 267 encoding CYP450.

Gene No.	Protein ID	Function	Contig ID	Gene Localization	Size, bp
1	fig|1827.715.peg.15	Cytochrome P450	NZ_MRBQ01000001.1	13,731–14,894	1163
2	fig|1827.715.peg.264	Putative cytochrome P450	NZ_MRBQ01000001.1	296,335–297,735	1400
3	fig|1827.715.peg.4576	Putative cytochrome P450 hydroxylase	NZ_MRBQ01000022.1	12,677–14,065	1388
4	fig|1827.715.peg.4821	Putative cytochrome P450 hydroxylase	NZ_MRBQ01000024.1	45,187–46,440	1253
5	fig|1827.715.peg.6087	Cytochrome P450	NZ_MRBQ01000041.1	52,406–53,776	1370
6	fig|1827.715.peg.6331	Putative cytochrome P450 hydroxylase	NZ_MRBQ01000046.1	28,292–29,515	1223
7	fig|1827.715.peg.6960	Cytochrome P450 monooxygenase	NZ_MRBQ01000085.1	1888–3099	1211

**Table 5:** Genetic surroundings of genes encoding CYP450 enzymes in *R*. *qingshengii* IEGM 267.

Gene No.	Upstream Transcriptional Regulators	Proteins Participating in Electron Transfers and Redox Reactions	Mobile Elements and Transposases	Other
1	AcrR family	Dehydrogenases with different specificities (related to short-chain alcohol dehydrogenases), Glyoxalase/bleomycin resistance protein/dioxygenase	–	–
2	AcrR family	3-ketoacyl-CoA thiolase (EC 2.3.1.16) @ Acetyl-CoA acetyltransferase (EC 2.3.1.9)	–	Transcriptional regulator, AcrR family
3	AcrR family	Short-chain dehydrogenase, Ferredoxin reductase, Ferredoxin, 2Fe-2S	–	Uncharacterized MFS-type transporter, Transcriptional regulator, AraC family
4	–	L-carnitine dehydratase/bile acid-inducible protein F, Oxidoreductase, short-chain dehydrogenase/reductase family, conserved protein associated with acetyl-CoA C-acyltransferase, Isochorismatase (EC 3.3.2.1), 2 oxidoreductases, short-chain dehydrogenase/reductase family	–	Transcriptional regulator, AraC family
5	AcrR family	Hypotetical proteins	–	Possible restriction/modification enzyme, DNA/RNA helicases, SNF2 family, Helicase, C-terminal:Type III restriction enzyme, res subunit:DEAD/DEAH box helicase, N-terminal
6	HxlR family	Nitroreductase, 4 ABC transporters, Trans-aconitate 2-methyltransferase (EC 2.1.1.144), Long-chain-fatty-acid–CoA ligase (EC 6.2.1.3), Lipase 1 (EC 3.1.1.3)	–	–
7	MerR family	Arsenite/antimonite pump-driving ATPase ArsA (EC 3.6.3.16), Asenic metallochaperone ArsD, transfers trivalent metalloids to ArsAB pump, Arsenate-mycothiol transferase (EC 2.8.4.2), Thioredoxin reductase (EC 1.8.1.9), possible ethyl tert-butyl ether degradation protein, 2-polyprenylphenol hydroxylase and related flavodoxin oxidoreductases/CDP-6-deoxy-delta-3,4-glucoseen reductase-like	Mobile element protein	Transcriptional regulator, AraC family

**Table 6:** Homology of CYP450 among UniProt dataset.

Entry	Protein Name	Organism	Length, AA	Gene Ontology	Identity, %
No. 1 (fig|1827.715.peg.15)					
A0AB38RFR6	Cytochrome P450	*Rhodococcus qingshengii* JCM 15477	411	cholest-4-en-3-one 26-monooxygenase activity, heme binding, iron ion binding, steroid hydroxylase activity, cholesterol catabolic process	99.7
A0ACD7JB72		*Rhodococcus erythropolis* R138	411	ND *	98.4
C0ZRV1		*Rhodococcus erythropolis* (strain PR4/NBRC 100887)	411	cholest-4-en-3-one 26-monooxygenase activity, heme binding, iron ion binding, steroid hydroxylase activity, cholesterol catabolic process	98.4
A0ABV5XL44		*Rhodococcus baikonurensis*	410		97.2
A0ABU4BRC7		*Rhodococcus globerulus*	410		92.4
No. 2 (fig|1827.715.peg.264)					
A0AB38RF50	Cytochrome P450	*Rhodococcus qingshengii* JCM 15477	466	heme binding, iron ion binding, monooxygenase activity, oxidoreductase activity, acting on paired donors, with incorporation or reduction of molecular oxygen	99.6
C0ZQC6		*Rhodococcus erythropolis* (strain PR4/NBRC 100887)	466		99.6
A0ACD7JCB7		*Rhodococcus erythropolis* R138	466	ND *	99.1
A0ABV5XNA9		*Rhodococcus baikonurensis*	466	heme binding, iron ion binding, monooxygenase activity, oxidoreductase activity, acting on paired donors, with incorporation or reduction of molecular oxygen	98.5
No. 3 (fig|1827.715.peg.4576)					
A0A177YEZ6	Cytochrome	*Rhodococcoides kyotonense*	462	heme binding, iron ion binding, monooxygenase activity, oxidoreductase activity, acting on paired donors, with incorporation or reduction of molecular oxygen	100
A0ABU4D8J4	Cytochrome P450	*Rhodococcus cerastii*	462		100
A0A143QFC8	Linalool 8-monooxygenase, EC:1.14.13.151	*Rhodococcus fascians*	462		100
A0ABU9CV92	Cytochrome P450	*Rhodococcoides navarretei*	462		100
A0ABU4B4Q1		*Rhodococcus cercidiphylli*	462		99.1
A0ABU4F113		*Williamsia marianensis*	462		99.1
A0A2S2C862		*Rhodococcus oxybenzonivorans*	462		94.6
A0A2S0KG06		*Gordonia iterans*	462		92
No. 4 (fig|1827.715.peg.4821)					
A0ABW6SET8	Cytochrome P450	*Nocardia jiangxiensis*	417	cholest-4-en-3-one 26-monooxygenase activity, heme binding, iron ion binding, steroid hydroxylase activity, cholesterol catabolic process	88
A0ABT6BWC5		*Gordonia hongkongensis*	421		76.1
A0ABU4EYX5		*Williamsia marianensis*	421		75.4
No. 5 (fig|1827.715.peg.6087)					
A0AB38RGW7	Cytochrome P450	*Rhodococcus qingshengii* JCM 15477	456	heme binding, iron ion binding, monooxygenase activity, oxidoreductase activity, acting on paired donors, with incorporation or reduction of molecular oxygen, sterol metabolic process	100
A0AAX3YGM1		*Rhodococcus opacus*	453		85.7
A0ABU4BVX6		*Rhodococcus globerulus*	455		84.3
A0ACD7J0N5		*Rhodococcus erythropolis* R138	455	ND	85
C0ZQE3		*Rhodococcus erythropolis* (strain PR4/NBRC 100887)	455	heme binding, iron ion binding, monooxygenase activity, oxidoreductase activity, acting on paired donors, with incorporation or reduction of molecular oxygen, sterol metabolic process	84.8
No. 6 (fig|1827.715.peg.6331)					
A0AB38RBK8	Cytochrome P450	*Rhodococcus qingshengii* JCM 15477	407	cholest-4-en-3-one 26-monooxygenase activity, heme binding, iron ion binding, steroid hydroxylase activity, cholesterol catabolic process	99.3
A0ABV5XHJ0		*Rhodococcus baikonurensis*	413		92.4
C0ZPE5		*Rhodococcus erythropolis* (strain PR4/NBRC 100887)	407		92.4
A0ACD7J150		*Rhodococcus erythropolis* R138	407	ND	91.9
A0A163L6B6	Heme binding	*Didymella rabiei* (Chickpea ascochyta blight fungus) (*Mycosphaerella rabiei*)	328	cholest-4-en-3-one 26-monooxygenase activity, heme binding, iron ion binding, steroid hydroxylase activity, cholesterol catabolic process	98.5
No. 7 (fig|1827.715.peg.6960)					
A0A318RX24	Cytochrome P450	*Williamsia limnetica*	403	heme binding, iron ion binding, monooxygenase activity, oxidoreductase activity, acting on paired donors, with incorporation or reduction of molecular oxygen	94.8
A0ABT4MS37		*Gordonia rubripertincta*	403		94.8
A0ABU2GUN3		*Gordonia westfalica*	403		91.8
A0AAX3T7H3		*Gordonia hongkongensis*	403		92.1
A0ABQ0HDM6		*Gordonia terrae* NBRC 100016	403		92.1
A0ABQ0HMM7		*Gordonia rubripertincta* NBRC 101908	403		91.3

* ND—no data.

## Data Availability

The data presented in this study are available on request from the corresponding author. The draft genome sequence data are available at NCBI under GenBank accession number MRBQ00000000.1.

## Supplementary Materials

The following supporting information can be downloaded at https://www.mdpi.com/article/10.3390/molecules31173083/s1. Figure S1. GC-MS chromatogram of sterile RS media with (–)-*trans*-carveol after 7 days of incubation (abiotic control); Figure S2: Percent identity matrix distribution for CYP Nos. 1–7 and CDH; Table S1. GC-MS data for samples after 7 days of incubation; Table S2: Differentiating physiological and biochemical properties of *Rhodococcus qingshengii* IEGM 267; Table S3: Strains in the dataset and their type-based species and subspecies clustering; Table S4: Results for CDH homolog search in the proteome of *R. qingshengii* IEGM 267.

## Abbreviations

The following abbreviations are used in this manuscript:

CDH	Carveol dehydrogenase
CDS	Coding sequence
CYP450	Cytochrome P450
GC-MS	Gas chromatography–mass spectrometry
*R*.	*Rhodococcus*
TLC	Thin-layer chromatography
