# Supplementary material for: Whole-Genome Sequence Dataset of Rhodococcus qingshengii IEGM 267—Terpenoid Biotransformer Toward Genetic Functional Annotation
Source: Molecules. 2026 Sep 2;31(17):3083. doi: 10.3390/molecules31173083 (PMC13566975; doi:10.3390/molecules31173083)
Supplement: Supplementary file 1 [file molecules-31-03083-s001.zip › molecules-4494246-supplementary.pdf]

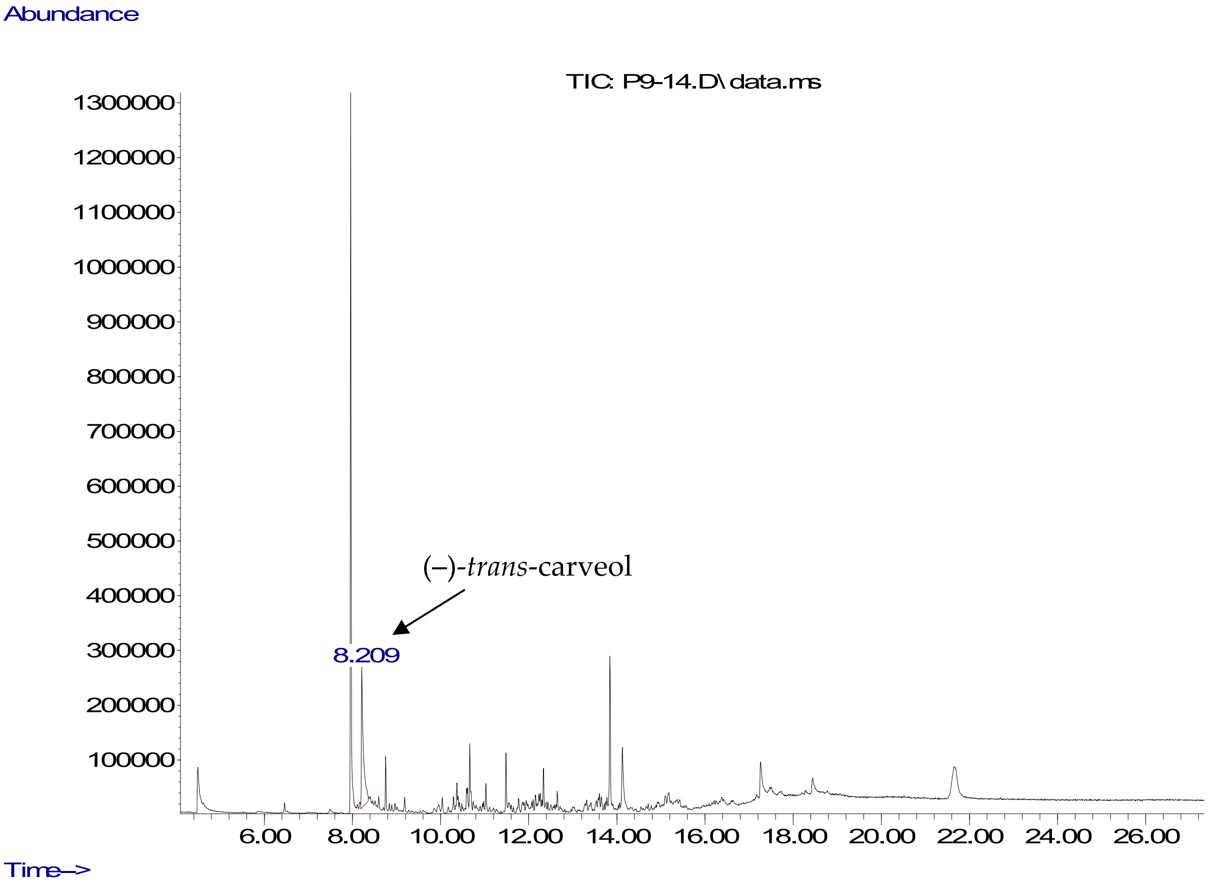

Figure S1. GC-MS chromatogram of sterile RS media with (–)-*trans*-carveol after 7 days of incubation (abiotic control).

Table S1. GC-MS data for samples after 7 days of incubation

peak #	R.T. min	first scan	max scan	last scan	PK TY	peak height	corr. area	corr. % max	% of total
Abiotic control									
1	8.209	560	563	583	rM	257569	803835	100	100
R. qingshengii IEGM 267 with (–)-*trans*-carveol									
1	8.209	561	563	570	rM	56560	128332	4.81	4.645
2	8.407	587	590	618	rM	985267	2634255	100	95.355

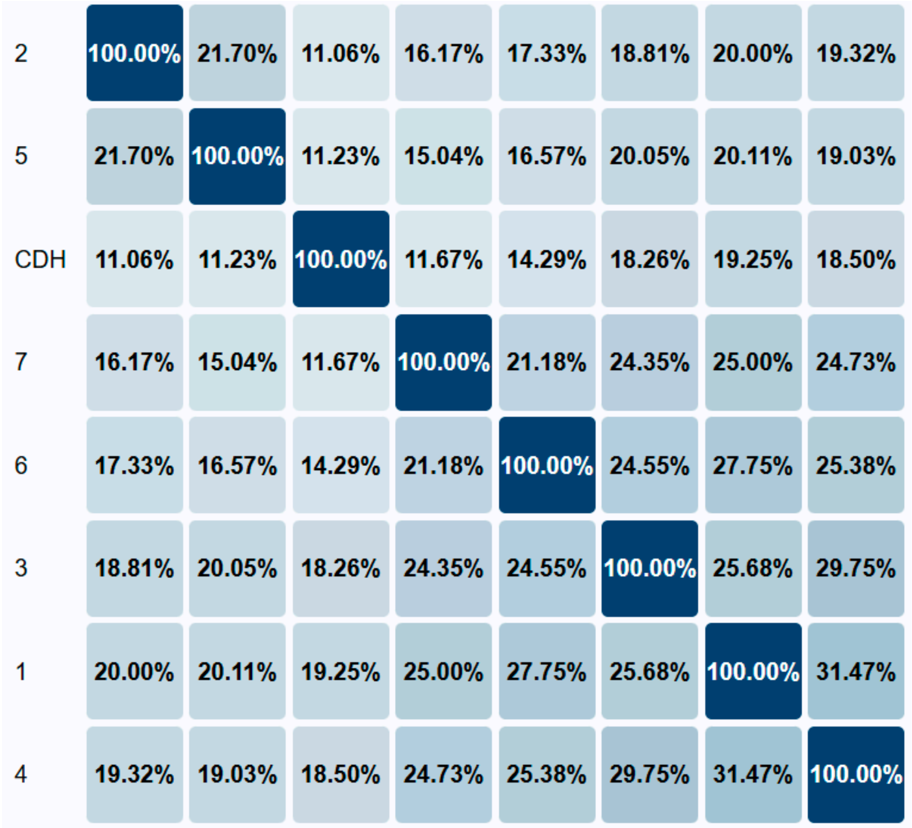

Figure S2. Percent identity matrix distribution for CYP Nos. 1–7 and CDH. Image obtained using Clustal2.1.

Table S2. Differentiating physiological and biochemical properties of *R. qingshengii* IEGM 267.

Feature	Manifestation (yes/no)
Urease activity	Yes
Catalase activity	Yes
The acid is formed from alpha-methyl-D-glucoside	No
The acid is formed from arabinose	No
The acid is formed from dulcitol	No
The acid is formed from inositol	Yes
The acid is formed from inulin	No
The acid is formed from sucrose	Yes
The acid is formed from sorbitol	No
The acid is formed from xylose	No
The acid is formed from lactose	No
The acid is formed from trehalose	Yes
The acid is formed from maltose	No
The acid is formed from fructose	Yes
The acid is formed from raffinose	No
The acid is formed from myo-inositol	No
Lactic acid is used	No
Succinic acid is used	Yes

Table S3. Strains in the dataset and their type-based species and subspecies clustering.

TYGS ID	Kind	Species cluster	Subspecies cluster	Preferred name	Deposit	Authority	Other deposits	Synonymous taxon names	Bioproject accession	Biosample accession	Assembly accession
11710	type strain	1	0	*Antrihabitans cavernicola*	C1-24	(Lee et al. 2020) Val-Calvo and Vázquez-Boland 2024	DSM 109484; KACC 19964	*Antrihabitans cavernicola*; *Rhodococcus cavernicola*; *Spelaeibacter cavernicola*	PRJNA555190	SAMN12303457	GCA_008297975
148796	type strain	2	1	*Rhodococcus baikonurensis*	JCM 11411	Li et al. 2004	A1-22; DSM 44587; NBRC 100611; CCM 8450; GTC 1041	*Rhodococcus baikonurensis*	PRJDB10510	SAMD00645509	GCA_039535945
157825	type strain	3	3	*Rhodococcus erythropolis*	JCM 3201	(Gray and Thornton 1928) Goodfellow and Alderson 1979 emend. Nouioui et al. 2018	CIP 104179; NRRL B-16025; NCIMB 9158; ATCC 4277; DSM 43066; JCM 20419; JCM 3201; IFO 15567; NBRC 15567; NCTC 13021; IEGM 7; VKM Ac-858; HAMBI 1953; LMG 5359; N11; NCIB 9158	*Mycobacterium erythropolis*; *Nocardia erythropolis*; *Rhodococcus erythropolis*	PRJDB7527	SAMD00143522	GCA_003990875
4055	type strain	3	3	*Rhodococcus erythropolis*	NBRC 15567	(Gray and Thornton 1928) Goodfellow and Alderson 1979 emend. Nouioui et al. 2018	CIP 104179; NRRL B-16025; NCIMB 9158; ATCC 4277; DSM 43066; JCM 20419; JCM 3201; IFO 15567; NBRC 15567; NCTC 13021; IEGM 7; VKM Ac-858; HAMBI 1953;	*Mycobacterium erythropolis*; *Nocardia erythropolis*; *Rhodococcus erythropolis*	PRJDB293	SAMD00046519	GCA_001552595

							LMG 5359; N11; NCIB 9158				
13730	type strain	4	2	*Rhodococcus globerulus*	NBRC 14531	Goodfellow et al. 1985 emend. Nouioui et al. 2018	CIP 104174; NRRL B-16938; ATCC 25714; DSM 43954; JCM 7472; IFO 14531; IEGM 591; R58	*Rhodococcus globerulus*	PRJDB295	SAMD00047211	GCA_001894805
20318	type strain	4	2	*Nocardia globerula*	DSM 44596	(Gray 1928) Waksman and Henrici 1948	ATCC 19370; JCM 7475	*Mycobacterium globerulum*; *Nocardia globerula*; *Proactinomyces globerulus*	PRJNA553792	SAMN12250501	GCA_008125105
5298	type strain	5	4	*Rhodococcus opacus*	DSM 43205	Klatte et al. 1995	CIP 104549; ATCC 51881; DSM 43205; JCM 9703; IFO 16217; NBRC 16217; NBRC 100624	*Rhodococcus opacus*	PRJNA224116	SAMN04357312	GCF_001646735
157865	type strain	6	7	*Rhodococcus enclensis*	NIO-1009	Dastager et al. 2014 emend. Nouioui et al. 2018	DSM 45688; NCIM 5452; NIO-1009	*Rhodococcus enclensis*	PRJEB15019	SAMN04487806	GCA_900094765
20182	type strain	6	7	*Rhodococcus jialingiae*	djl-6-2	Wang et al. 2010	DSM 45257; JCM 16906; CCTCC AB 208292	*Rhodococcus jialingiae*	PRJNA429114	SAMN08329308	GCA_002893965
3771	type strain	6	7	*Rhodococcus enclensis*	DSM 45688	Dastager et al. 2014 emend. Nouioui et al. 2018	DSM 45688; NCIM 5452; NIO-1009	*Rhodococcus enclensis*	PRJNA302094	SAMN04263893	GCA_001456965
5384	type strain	6	7	*Rhodococcus qingshengii*	JCM 15477	Xu et al. 2007 emend. Nouioui et al. 2018	CGMCC 1.6580; KCTC 19205; DSM 45222; djl-6	*Rhodococcus qingshengii*	PRJNA224116	SAMN04357315	GCF_001646745

U1227471	user strain	6	7	PRJNA356206							
65282	type strain	7	5	'*Rhodococcus tibetensis*'*	FXJ9.536	Huang et al. 2022	CGMCC 4.7853	*Rhodococcus tibetensis*	PRJNA859995	SAMN29814303	GCA_024438035
8646	type strain	8	6	'*Rhodococcus mengyinensis*'*	NM-2T	Wei et al. 2025	CGMCC 1.61589; JCM 36291	*Rhodococcus mengyinensis*	PRJNA1226358	SAMN46927280	GCA_048381435

* - Not a validly published name

Table S4. Results for CDH homologs search in the proteome of *R. qingshengii* IEGM 267.

№	Cluster representative sequence	Max Score	Total Score	Query Covery	E value	Per. Ident.	Acc. Len
1	WP_042452217.1 MULTISPECIES: mycofactocin-coupled SDR family oxidoreductase [Rhodococcus erythropolis group]	303	303	100%	1e-104	56.88%	276
2	WP_020970454.1 MULTISPECIES: mycofactocin-coupled SDR family oxidoreductase [Rhodococcus]	275	275	100%	1e-93	52.71%	281
3	WP_047269493.1 mycofactocin-coupled SDR family oxidoreductase [Rhodococcus erythropolis]	251	251	100%	2e-84	49.46%	265
4	WP_073512545.1 mycofactocin-coupled SDR family oxidoreductase [Rhodococcus erythropolis]	242	242	99%	9e-81	46.98%	274
5	WP_073513227.1 mycofactocin-coupled SDR family oxidoreductase [Rhodococcus erythropolis]	241	241	99%	3e-80	48.06%	287
6	WP_047269630.1 MULTISPECIES: mycofactocin-coupled SDR family oxidoreductase [Rhodococcus]	214	214	99%	2e-69	42.34%	282
7	WP_030536568.1 MULTISPECIES: mycofactocin-coupled SDR family oxidoreductase [Rhodococcus]	211	211	98%	2e-68	43.75%	271
8	WP_047270120.1 MULTISPECIES: mycofactocin-coupled SDR family oxidoreductase [Rhodococcus]	208	208	100%	2e-67	41.67%	270
9	WP_007728643.1 MULTISPECIES: mycofactocin-coupled SDR family oxidoreductase [Rhodococcus]	208	208	100%	3e-67	41.32%	285
10	WP_003941966.1 MULTISPECIES: mycofactocin-coupled SDR family oxidoreductase [Rhodococcus]	203	203	98%	2e-65	41.88%	275
